# Editorial: Microbial Siderophores: Biosynthesis, Regulation, and Physiological and Ecological Impacts

**DOI:** 10.3389/fmicb.2022.892485

**Published:** 2022-04-18

**Authors:** Haichun Gao, Xiaoying Bian

**Affiliations:** ^1^Institute of Microbiology, College of Life Sciences, Zhejiang University, Hangzhou, China; ^2^Helmholtz International Lab for Anti-infectives, Shandong University-Helmholtz Institute of Biotechnology, State Key Laboratory of Microbial Technology, Shandong University, Qingdao, China

**Keywords:** siderophore, siderophore biosynthesis, iron uptake, bacteria, iron homeostasis

Iron is essential for virtually all living organisms because iron-dependent proteins are employed to perform a myriad of functions in diverse biological processes (Wandersman and Delepelaire, 2004; Andrews et al., 2013). Siderophore-dependent iron acquisition is the most efficient strategy for microorganisms to obtain iron from the low-iron environments (Wilson et al., 2016). Siderophores are small molecules specifically chelating ferric ions and are categorized into several groups based on the iron-binding moiety. Siderophores are generally inducibly synthesized and secreted in response to iron starvation (Andrews et al., 2013; Chareyre and Mandin, 2018). While the diversity of the siderophore biosynthetic pathways is enormous, they either employ non-ribosomal peptide synthetases (NRPSs) or NRPS-independent enzymes (Barry and Challis, 2009). Microorganisms usually produce multiple different siderophores, with one primarily responsible for iron acquisition and others implicated in a variety of physiological processes (Rütschlin et al., 2018). Many siderophores possess beneficial and antimicrobial properties, playing an important role in directly shaping microbial community by mediating cooperative, exploitative and competitive interactions (Kramer et al., 2020). Moreover, recently they show potential in medical and environmental applications (Kurth et al., 2016; Ribeiro and Simões, 2019).

This Research Topic contains four original research and one review articles. Two articles discuss the physiological function of siderophores produced by *Yersinia pseudotuberculosis* and *Salmonella enteritidis*. It has been well known that an *iucABCD*-*iutA* operon encodes a biosynthesis system for aerobactin in many bacteria. But this may not be the case in *iucABCD*-*iutA*-carrying pathogenic *Yersinia* spp. as aerobactin had never been detected before the study of Li et al. In their article, multiple lines of evidence were presented to show that *Y. pseudotuberculosis* YPIII is able to produce aerobactin, which is fully dependent on the operon. The operon appears to be under the direct repression of the ferric uptake regulator (Fur) in response to changes in iron concentrations. This aerobactin-mediated iron acquisition system not only plays an important role in supporting growth under low-iron conditions, but also is involved in biofilm formation, resistance to oxidative stress, removal of reactive oxygen species, and virulence. The work emphasizes the importance of aerobactin in the general physiology and the pathogenesis of *Y. pseudotuberculosis*. The investigations carried out by Wellawa et al. focus on the role of the siderophore-mediated (enterobactin and salmochelin) Fe^3+^ and the FeoABC-mediated Fe^2+^ uptake systems in colonization of *Salmonella enteritidis* in chicken. The results clearly show that both systems contribute in cecal colonization, rapid systemic spread, and survival in extraintestinal sites in chickens. By using a bioluminescent reporter, the authors were able to visualize the changes in iron availability during gastrointestinal colonization of *Salmonella via ex vivo* imaging. They found that in the cecal compartment iron shortage becomes apparent to the bacterial cells even at early colonization stages. To overcome this, both iron acquisition systems are required although there is a redundancy. Overall, the work provides an invaluable insight into the impacts of iron acquisition on Salmonella colonization in chickens and stresses the need for an understanding of *Salmonella* iron homeostasis and its regulation.

A research article by Zhang et al. aims at siderophore biosynthesis in fungi, in which these small molecular iron chelators participate in the multiple cellular processes. They discovered the siderophores and identified their biosynthetic pathway from *Metarhizium robertsii* ARSEF2575. This study highlights the method using differentially transcriptional expressions of biosynthetic genes under different iron concentration conditions. It was found that three genes from different NRPS gene clusters were upregulated under iron-deficient conditions, which leads to the identification of new coprogen metachelin C that was connected to a gene cluster by deletion of *mrsidA* and *mrsidD*. This work lays a foundation for further finding for new siderophores and studying the functions of siderophores. Apart from this, a research article by Khan et al. discusses activity of catecholate siderophores released by *E. coli* against fungus *Aspergillus nidulans*. The study found that catecholate siderophore purified from an *E. coli* strain, presumably enterobactin, impacts the physiology of *A. nidulans* profoundly, evidenced by decreased colony size, increased filament length, and altered hyphal branching pattern. Interestingly, siderophore-treated cells show a reduction in the overall antioxidative enzyme activity but catalase appears to be different. Further analyses reveal that upon siderophore exposure, the fungal cells suffer from the membrane damage judged by changed malondialdehyde contents. Authors proposed that the oxidative stress caused by siderophore-mediated iron influx largely accounts for the inhibition and killing.

The only review article by Liu et al. consider recent advances in the siderophore biology of *Shewanella*, a group of ubiquitously distributed γ-proteobacteria that include animal/human pathogens and ones well-recognized for their potential in bioenergy. These bacteria encode and produce a large number of iron-containing proteins, cytochromes *c* in particular, and therefore, have a high demand for iron. To meet this, an array of novel features have been evolved, including a siderophore system that is able to synthesize a variety of siderophores, many siderophore receptors that recognize siderophores released by other bacteria, regulatory systems that coordinate iron uptake, storage, and assumption for maintaining homeostasis. They also review physiological impacts of siderophore in these bacteria, some of which are unusual, especially those on cytochrome *c* biosynthesis. Although to support growth the siderophore-mediated Fe^3+^ uptake system is still secondary to the feo-mediated Fe^2+^ uptake system, the former is closely linked to cytochrome *c* biosynthesis. Given cytochromes *c* endow *Shewanella* respiratory versatility and great potential for biotechnological applications, the biology of siderophore in these bacteria appears to be particularly important and needs to be explored further as the current understanding of the subject in *Shewanlle* is rather limited.

The articles in this Research Topic shed light on the diverse roles of siderophores for physiological adaptation to intra/extracellular environmental change. We hope that the findings presented in these topic articles will pave the way for future studies on the biochemistry, physiology, ecology, and medical and environmental applications of microbial siderophores.

## Author Contributions

Both authors listed have made a substantial, direct, and intellectual contribution to the work and approved it for publication.

## Funding

This research was supported by National Natural Science Foundation of China, 41976087 and 31930003.

## Conflict of Interest

The authors declare that the research was conducted in the absence of any commercial or financial relationships that could be construed as a potential conflict of interest.

## Publisher's Note

All claims expressed in this article are solely those of the authors and do not necessarily represent those of their affiliated organizations, or those of the publisher, the editors and the reviewers. Any product that may be evaluated in this article, or claim that may be made by its manufacturer, is not guaranteed or endorsed by the publisher.
